# Effects of Replacing Fish Meal with Poultry By-Product Meal on Growth, Physiological Status, Disease Resistance, and Hepatic Transcriptomic Responses in Hybrid Yellow Catfish (*Pelteobagrus fulvidraco* ♀ × *Pelteobagrus vachelli* ♂)

**DOI:** 10.3390/metabo16090694

**Published:** 2026-09-19

**Authors:** Peng Shi, Lei Wang, Xuxiong Huang, Biao Yun, Xueqiao Qian

**Affiliations:** 1 Animal Husbandry and Fisheries Research Center of Guangdong Haid Group Co., Ltd., Guangzhou 511400, China; 2 Guangdong Hairui Biotechnology Co., Ltd., Guangzhou 511400, China; 3 College of Fisheries and Life Science, Shanghai Ocean University, Shanghai 201306, China

**Keywords:** hybrid yellow catfish, poultry by-product meal, fish meal, growth performance, antioxidant capacity, immune response

## Abstract

Purpose: This study evaluated the effects of replacing dietary fish meal (FM) with poultry by-product meal (PBM) on growth, physiological status, disease resistance, and hepatic transcriptomic responses in hybrid yellow catfish (*Pelteobagrus fulvidraco* ♀ × *Pelteobagrus vachelli* ♂). Methods: Six isonitrogenous and isolipidic diets were formulated by replacing 0%, 12%, 24%, 36%, 48%, or 60% of dietary FM with PBM. Juveniles (initial weight 5.18 ± 0.02 g) were fed the experimental diets for 8 weeks. Results: Weight gain was maximized and the feed conversion ratio minimized in fish fed the FM19 diet, with broken-line regression analysis of weight gain estimating the optimal FM replacement level at 27.4%. Hepatic and intestinal superoxide dismutase activities, as well as intestinal trypsin activity, tended to decline as the level of FM replacement with PBM increased. Following *Edwardsiella ictaluri* challenge, 96-h cumulative survival was lower in the FM10 group than in all other groups. Principal component analysis of hepatic transcriptomes clearly separated the FM10 group from the FM25 and FM19 groups. Gene Ontology analysis associated differentially expressed genes with responses to hormones and endogenous stimuli (FM10 vs. FM25), protein folding and chaperone-mediated complex assembly (FM19 vs. FM25), and catabolic processes and mitochondrial function (FM10 vs. FM19). Kyoto Encyclopedia of Genes and Genomes analysis identified pathways involving mitogen-activated protein kinase signaling, protein processing in the endoplasmic reticulum, autophagy, apoptosis, and inflammatory responses. Moreover, genes associated with oxidative stress and inflammation showed higher expression in the FM10 group compared with the FM19 and FM25 groups. Conclusions: Moderate FM replacement with PBM improved growth and feed utilization, whereas excessive replacement was associated with lower antioxidant and digestive enzyme activities, altered metabolic responses, and enhanced expression of inflammation-related genes, supporting PBM as a partial FM substitute in low-FM diets for hybrid yellow catfish.

## 1. Introduction

Fish meal (FM) remains an important protein source in aquafeeds due to the high digestibility, amino acid profile, and palatability [1,2,3,4]. However, the FM supply has not increased at the same pace as global aquaculture production [5,6,7]. This imbalance has increased FM price volatility and the aquafeed production costs, raising concerns about the long-term sustainability of aquaculture [1,2]. Therefore, identifying nutritionally adequate, economically viable, and environmentally sustainable alternative protein sources has become a major research priority in aquaculture [2,3,4].

Poultry by-product meal (PBM) is a rendered animal protein ingredient typically produced from poultry processing by-products through grinding, cooking, defatting, and drying. Owing to the stable supply, relatively high protein content, and relatively favorable amino acid profile, PBM represents a promising terrestrial animal protein ingredient for partially replacing FM in aquafeeds [8,9,10]. Previous studies have demonstrated the potential of PBM to partially replace FM in diets for several aquaculture species, including cuneate drum (*Nibea miichthioides*), black sea turbot (*Scophthalmus maeoticus*), cobia (*Rachycentron canadum*), florida pompano (*Trachinotus carolinus*), yellow catfish (*Pelteobagrus fulvidraco*), and largemouth bass (*Micropterus salmoides*) [11,12,13,14,15,16]. Nevertheless, the optimal replacement level is species-specific, and extensive replacement of FM with PBM may induce amino acid imbalance, reduce digestive enzyme activity and feed palatability, and consequently impair growth and health [17,18,19]. Therefore, determining the appropriate PBM replacement level for individual fish species is essential to enable the precise formulation of low-FM aquafeeds.

Hybrid yellow catfish (*P. fulvidraco* ♀ × *P. vachelli* ♂) is an economically important hybrid produced through artificial breeding. Compared with its parental species, this hybrid exhibits pronounced heterosis, including superior growth performance, survival, and hypoxia tolerance [20,21]. Preliminary studies have explored alternative dietary protein sources for this hybrid. For example, a previous study reported that maggot meal could replace up to 40% of dietary FM without adversely affecting growth performance, body composition, or antioxidant capacity [22]. This finding suggests that alternative animal-protein ingredients may have potential as FM substitutes in this hybrid. Although PBM is an animal-protein ingredient with potential for FM replacement, its effects in hybrid yellow catfish have not been sufficiently characterized, and the optimal level of FM replacement with PBM remains unclear. Further investigation is therefore warranted to evaluate the suitability of PBM as a partial substitute for FM and to determine its effects on the growth performance, physiological status, and disease resistance of this hybrid.

Accordingly, six experimental diets were formulated by replacing 0–60% of dietary FM with PBM. An 8-week feeding trial was conducted to evaluate the effects of dietary PBM inclusion on growth, physiological status, and disease resistance in hybrid yellow catfish. Hepatic transcriptomic analysis was subsequently performed to characterize the molecular mechanisms underlying metabolic and inflammatory responses to high replacement of FM with PBM. This study provides a basis for optimizing PBM utilization and formulating nutritionally balanced low-FM diets for hybrid yellow catfish.

## 2. Materials and Methods

### 2.1. Experimental Diets

Six isonitrogenous and isolipidic diets were formulated by replacing 0%, 12%, 24%, 36%, 48%, or 60% of dietary FM with PBM, designated FM25, FM22, FM19, FM16, FM13, and FM10, respectively (Table 1). All ingredients were ground to pass through a 60-mesh sieve, weighed according to the formulations, and thoroughly mixed using the stepwise expansion method. The mixtures were processed into 1.0-mm pellets using the single-screw extruder. The diets were stored in a cool, well-ventilated area.

### 2.2. Fish and Feeding Management

Experimental fish were cultured in the Aquatic Research and Development Base of Guangdong Haid Group Co., Ltd. (Guangzhou, China). Juvenile hybrid yellow catfish were acclimated to the experimental conditions for 14 days. Following acclimation, healthy fish of uniform size and with no visible external injuries were selected and fasted for 24 h. A total of 2400 fish (initial weight 5.18 ± 0.02 g) were randomly distributed among 24 cylindrical tanks (350 L), with 100 fish per tank and four replicate tanks assigned to each of the six dietary treatments. The 8-week feeding trial was conducted in a flow-through culture system. Throughout the trial, water temperature, dissolved oxygen concentration, and pH were monitored daily and maintained at 26–28 °C, ≥6.0 mg/L, and 7.5–8.5, respectively. The fish were fed twice a day (7:00 a.m. and 6:00 p.m.) until apparent satiation. Mortality was monitored and recorded daily.

### 2.3. Sample Collection and Biochemical Analyses

At the end of the 8-week feeding trial, fish were fasted for 24 h before sampling. The number and total biomass of fish in each tank were recorded to calculate survival rate (SR), weight gain rate (WGR), specific growth rate (SGR), and feed conversion ratio (FCR). Fish were anesthetized with 0.02% MS-222 (Shanghai Reagent, Shanghai, China) before dissection. Three fish were randomly collected from each tank. Body weight and body length were measured, after which the liver and viscera were dissected and weighed. For each tank, the tissue samples collected from the three fish were pooled separately according to the subsequent analysis. The pooled tissues were used for biochemical analyses and hepatic transcriptomic analysis, with each pooled sample representing one tank-level biological replicate. Condition factor (CF), hepatosomatic index (HSI), and viscerosomatic index (VSI) were calculated, and the liver and intestine were sampled and stored at −80 °C for subsequent analysis. The calculation formulas were as follows:

Weight gain rate (WGR, %) = 100 × (W_2_ − W_1_)/W_1_

Specific growth rate (SGR, % day^−1^) = 100 × (Ln W_2_ − Ln W_1_)/56 days

Survival rate (SR, %) = 100 × N_2_/N_1_

Condition factor (CF, g cm^−3^) = 100 × (final body weight/final body length^3^)

Hepatosomatic index (HSI, %) = 100 × (liver weight/fish weight)

Viscerosomatic index (VSI, %) = 100 × (viscera weight/fish weight)

Feed conversion ratio (FCR) = D_in_/(W_2_ − W_1_)

where W_2_ is the final weight of fish per tank, W_1_ is the initial weight of fish per tank, N_2_ is the final number of fish per tank, N_1_ is the initial number of fish per tank, Din is the dry diet consumed per tank.

Serum levels of triglycerides (TG, Cat. No. A110-1-1), total cholesterol (TC, Cat. No. A111-1-1), alanine aminotransferase (ALT, Cat. No. C009-2-1), and aspartate aminotransferase (AST, Cat. No. C010-2-1) were determined. In addition, the activities of superoxide dismutase (SOD, Cat. No. A001-3-2) and catalase (CAT, Cat. No. A007-1-1), and malondialdehyde (MDA, Cat. No. A003-1-2) content were assessed in both liver and intestinal tissues. Intestinal digestive activity was evaluated by measuring the activities of lipase (LPS, Cat. No. A054-2-1), α-amylase (AMS, Cat. No. C016-1-2), and trypsin (Cat. No. A080-2-2). All biochemical indices were measured using commercial assay kits purchased from Nanjing Jiancheng Bioengineering Institute (Nanjing, China), following the manufacturer’s instructions.

### 2.4. Bacterial Challenge

After the 8-week feeding trial, fish that had been fed the experimental diets for the entire 8-week period were randomly selected from the four replicate tanks assigned to each dietary treatment for the *Edwardsiella ictaluri* challenge test. Sixty fish were selected per dietary treatment and randomly distributed into three challenge tanks, with 20 fish per tank. All challenge groups were maintained under identical water-quality and husbandry conditions after bacterial injection. Thus, the three challenge tanks per dietary treatment were considered the biological replicates for the challenge experiment. The *E. ictaluri* strain used in the present study was obtained from and preserved at the Animal and Aquatic Research Center of Guangdong Haid Group Co., Ltd. Fish were intraperitoneally injected with 0.1 mL of an *E. ictaluri* suspension at a concentration of 1.0 × 10^5^ CFU/mL per fish. Following injection, all fish were maintained under identical water-quality and husbandry conditions, and mortality was monitored at 6, 12, 24, 48, 72, and 96 h after challenge.

### 2.5. RNA Sequencing Analysis

Liver samples from fish fed the FM25, FM19, and FM10 diets were selected for transcriptome sequencing. For each of these dietary treatments, four replicate tanks were selected, and three fish were randomly sampled from each selected tank. Liver tissues from the three fish within each tank were pooled to generate one composite sample. RNA-seq data were analyzed using a reference-based transcriptomic pipeline. Raw reads were evaluated using FastQC and processed with fastp to remove adapter-containing sequences, low-quality bases, and reads containing ambiguous bases. The resulting clean reads were aligned to the *P. fulvidraco* reference genome (NCBI accession GCF_022655615.1) using HISAT2. Gene-level read counts were quantified against the corresponding reference gene annotation using featureCounts. The raw gene-level count matrix was used for differential expression analysis with DESeq2, whereas normalized expression values were used for sample correlation and principal component analyses. Gene identifiers and symbols were obtained from the reference annotation, and functional annotations were assigned using the NCBI, GO, and KEGG databases. Differentially expressed genes were defined as genes with an absolute log2 fold change greater than 1 and *p*-value < 0.05.

### 2.6. Statistical Analysis

Data were presented as means ± standard errors of the mean (SEM). The tank was considered the experimental unit for all growth performance, feed utilization, biochemical, antioxidant, and digestive enzyme analyses. Data normality and homogeneity of variance were assessed using the Shapiro–Wilk and Levene’s tests, respectively. Dietary treatment effects were analyzed by one-way analysis of variance (ANOVA), followed, when significant, by Tukey’s honestly significant difference test. Statistical analyses were performed using SPSS Statistics 20.0 (IBM Corp., Armonk, NY, USA), with significance set at *p* < 0.05.

## 3. Results

### 3.1. Survival, Growth Performance and Feed Efficiency

**Figure 1:**
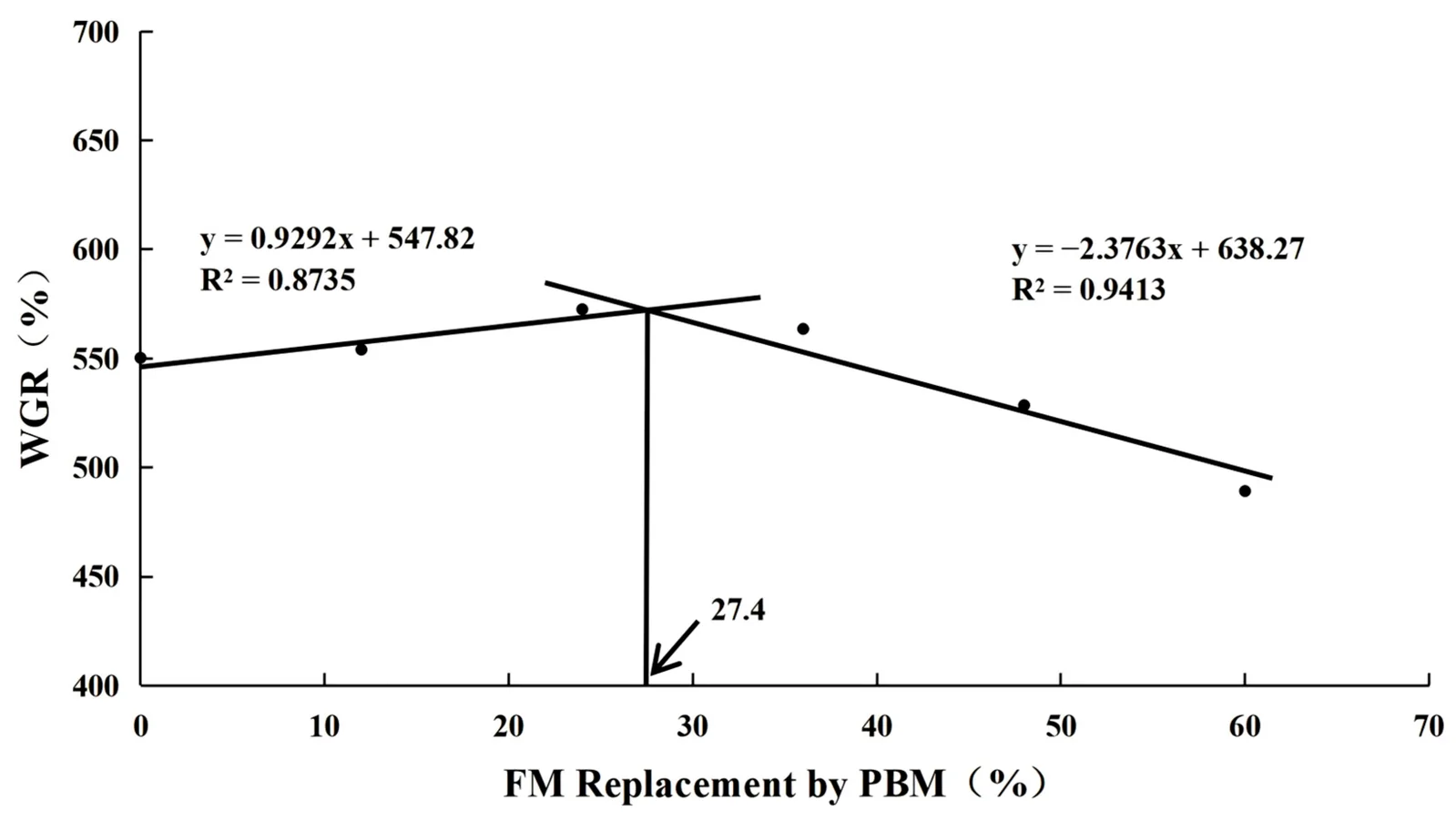
Broken-line model analysis of the relationship between the dietary FM replacement level with PBM and the WGR of hybrid yellow catfish (*P. fulvidraco* ♀ × *P. vachelli* ♂).The points represent the observed mean WGR values for the six dietary treatments, and the two oblique lines represent the fitted linear regression segments. The vertical line and arrow indicate the estimated breakpoint, corresponding to the optimal FM replacement level.

As shown in Table 2, SR did not differ significantly among the dietary treatments, except in the FM13 group. WGR and SGR increased from the FM25 group to the FM19 group, and then declined as FM replacement increased further. Both indices were highest in the FM19 group and lowest in the FM10 group (*p* < 0.05). FCR was significantly lower in the FM19 and FM16 groups than in the other groups (*p* < 0.05). The CF values were significantly higher in the FM25 and FM22 groups than in the FM16 and FM10 groups (*p* < 0.05). No significant differences were observed among treatments in HSI or VSI (*p* > 0.05). Broken-line regression based on WGR produced an estimated optimum FM replacement level of 27.4% (Figure 1).

### 3.2. Serum Biochemical Indices

Results showed that serum ALT and AST activities did not differ significantly among the dietary treatments (*p* > 0.05). Serum TG levels were significantly higher in the FM22, FM16, and FM10 groups than in the FM25 and FM13 groups (*p* < 0.05), whereas the FM19 group showed no significant difference from any of the other groups (*p* > 0.05). Serum TC concentration was significantly higher in the FM16 group than in the FM25, FM13, and FM10 groups (*p* < 0.05) (Table 3).

### 3.3. Hepatic and Intestinal Antioxidant Capacity

Results showed that Hepatic MDA content did not differ among dietary treatments (*p* > 0.05). Hepatic SOD activity showed a decreasing trend as the proportion of FM replaced by PBM increased. The FM25 group exhibited significantly higher hepatic SOD activity than the FM10 group (*p* < 0.05), while no significant differences were observed among the other groups (*p* > 0.05). Hepatic CAT activity differed significantly among treatments, reaching its highest level in the FM19 group, which was significantly higher than that in the FM22 group (*p* < 0.05) (Table 4).

Intestinal CAT activity did not differ significantly among the groups (*p* > 0.05). Intestinal SOD activity generally decreased with increasing replacement of FM by PBM. The FM25 group showed significantly higher intestinal SOD activity than the FM19, FM13, and FM10 groups (*p* < 0.05), with the lowest activity observed in the FM10 group. No significant difference in intestinal MDA content was found among the treatments (*p* > 0.05) (Table 4).

### 3.4. Intestinal Digestive Enzymes

No significant differences were observed in intestinal LPS or AMS activity among the groups (*p* > 0.05). In contrast, intestinal trypsin activity generally decreased with increasing replacement of FM with PBM. Trypsin activity was significantly higher in the FM25 group than in the FM19, FM16, FM13, and FM10 groups (*p* < 0.05), but did not differ significantly from that in the FM22 group (*p* > 0.05) (Table 5).

### 3.5. Post-Challenge Cumulative Survival

All groups showed cumulative survival rates above 95% at 6 h, with no significant differences among treatments. From 12 h onward, cumulative survival declined most rapidly in the FM10 group. At 12 h, the FM10 group was significantly lower than the FM25, FM22, FM19, FM16, and FM13 groups (*p* < 0.05). At 24 h, it was significantly lower than the FM25, FM22, FM19, and FM13 groups, but not the FM16 group. At 48 and 72 h, a significant difference was observed only between the FM10 and FM25 groups. At 96 h, the FM25 group had the highest cumulative survival rate, whereas the FM10 group had the lowest; the FM10 group was significantly lower than the FM25, FM22, FM19, and FM16 groups (*p* < 0.05), but did not differ significantly from the FM13 group (*p* > 0.05) (Table 6).

### 3.6. Hepatic Transcriptome Analysis

#### 3.6.1. Quality Control and Principal Component Analysis (PCA)

**Figure 2:**
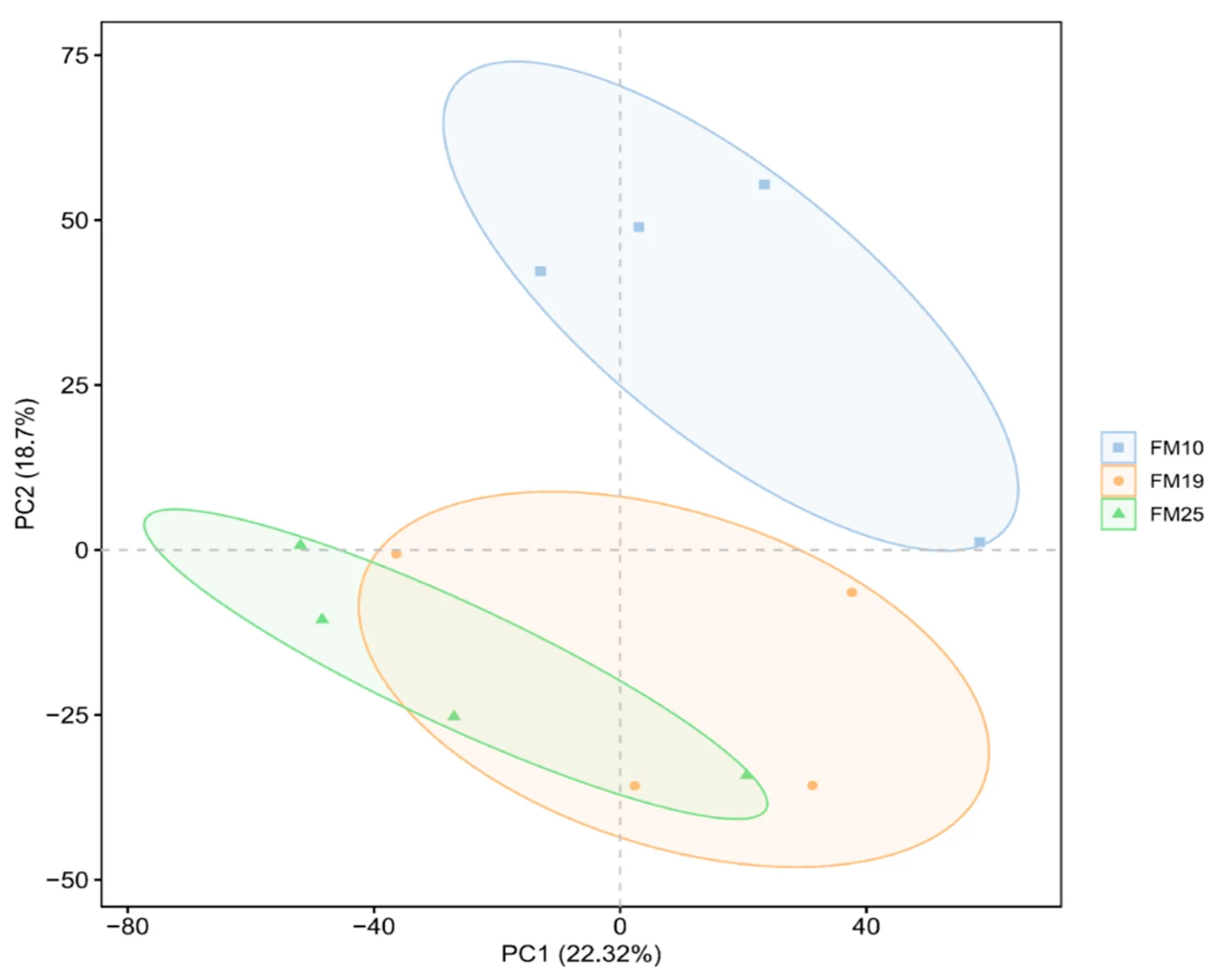
Principal component analysis score plot of samples from hepatic transcriptome in hybrid yellow catfish (*P. fulvidraco* ♀ × *P. vachelli* ♂). FM10, FM19, and FM25 represent the three dietary treatment groups. Each symbol represents an individual sample, and the ellipses indicate the distribution ranges of the corresponding groups. The gray dotted horizontal and vertical lines indicate zero scores on PC2 and PC1, respectively, and are included as reference lines.

Liver transcriptomes of hybrid yellow catfish were sequenced using the Illumina NovaSeq 6000 platform. A total of 87.98 Gb of clean bases were generated from 12 samples. Across all samples, Q20 and Q30 scores were at least 99.28% and 97.36%, respectively, while GC content ranged from 46.44% to 47.27% (Table 7). The PCA results showed that PC1 and PC2 explained 22.32% and 18.70% of the variation in gene expression, respectively, accounting for a cumulative 41.02% of the total variance. The confidence ellipse of the FM10 group was completely separated from those of the FM25 and FM19 groups (Figure 2).

#### 3.6.2. Differentially Expressed Gene (DEG) Analysis

**Figure 3:**
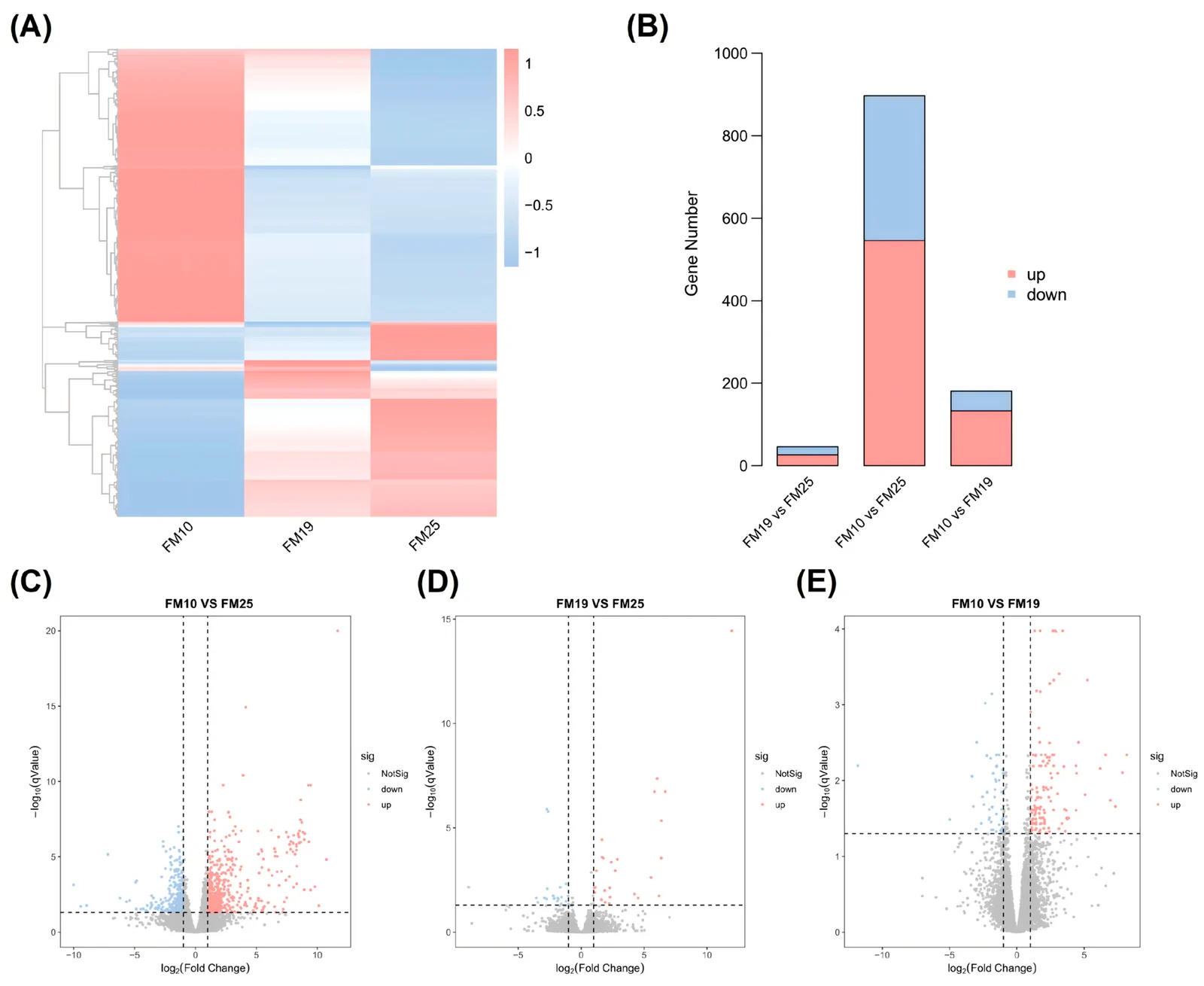
Distribution of DEGs in the hepatic transcriptome of hybrid yellow catfish (*P. fulvidraco* ♀ × *P. vachelli* ♂) fed FM10, FM19, and FM25 diets. (**A**) Hierarchical clustering heatmap of DEGs among the three dietary groups. (**B**) Numbers of upregulated and downregulated DEGs in the pairwise comparisons FM10 vs. FM25, FM19 vs. FM25, and FM10 vs. FM19. (**C**–**E**) Volcano plots of DEGs for FM10 vs. FM25 (**C**), FM19 vs. FM25 (**D**), and FM10 vs. FM19 (**E**). Red and blue dots represent significantly upregulated and downregulated genes, respectively, whereas gray dots indicate genes with no significant differential expression. Vertical and horizontal dashed lines indicate the thresholds for fold change and statistical significance.

DESeq2 analysis identified 897 DEGs in FM10 vs. FM25, comprising 546 upregulated and 351 downregulated genes; 46 DEGs in FM19 vs. FM25, comprising 26 upregulated and 20 downregulated genes; and 181 DEGs in FM10 vs. FM19, comprising 133 upregulated and 48 downregulated genes (Figure 3C–E).

**Figure 4:**
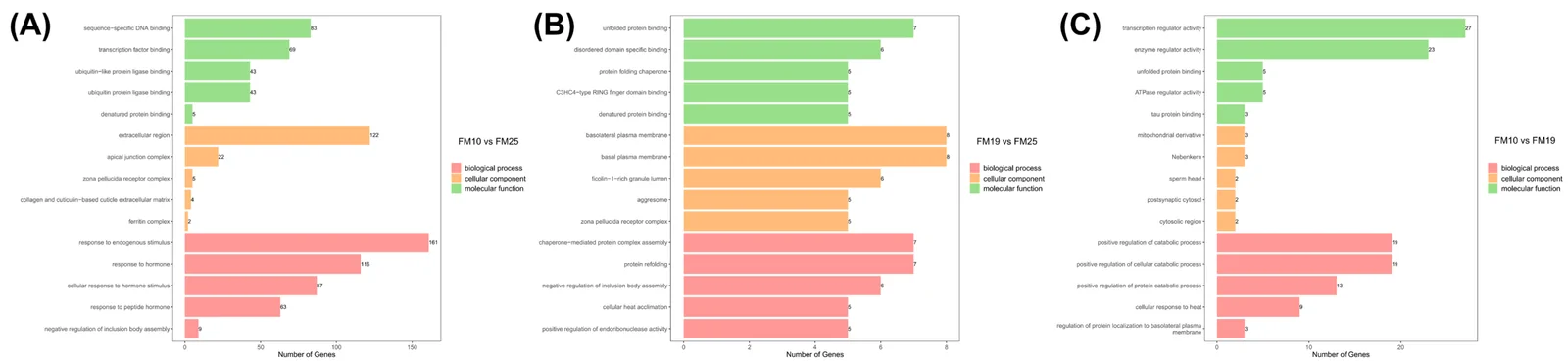
Gene Ontology enrichment analysis of DEGs in the hepatic transcriptome of hybrid yellow catfish (*P. fulvidraco* ♀ × *P. vachelli* ♂) fed FM10, FM19, and FM25 diets. (**A**–**C**) Significantly enriched GO terms for DEGs in the FM10 vs. FM25 (**A**), FM19 vs. FM25 (**B**), and FM10 vs. FM19 (**C**) comparisons. GO terms were classified into three major categories: biological process (red), cellular component (orange), and molecular function (green). The horizontal axis indicates the number of DEGs assigned to each GO term, and the vertical axis lists the enriched GO terms. Numbers at the ends of the bars indicate the corresponding DEG counts.

Gene Ontology (GO) analysis was performed on DEGs from three pairwise comparisons, and categorized into biological process (BP), cellular component (CC), and molecular function (MF). In FM10 vs. FM25, DEGs were primarily enriched in responses to endogenous stimuli and hormones (BP), the extracellular region and apical junction complex (CC), and sequence-specific DNA binding and transcription factor binding (MF). In FM19 vs. FM25, the primary enriched terms were associated with protein folding and chaperone-mediated protein complex assembly (BP), the basal and basolateral plasma membranes (CC), and unfolded protein and chaperone binding (MF). In FM10 vs. FM19, DEGs were mainly enriched in the positive regulation of catabolic processes (BP), mitochondrial derivatives and nucleosomes (CC), and transcriptional and enzymatic regulator activities (MF) (Figure 4A–C).

**Figure 5:**
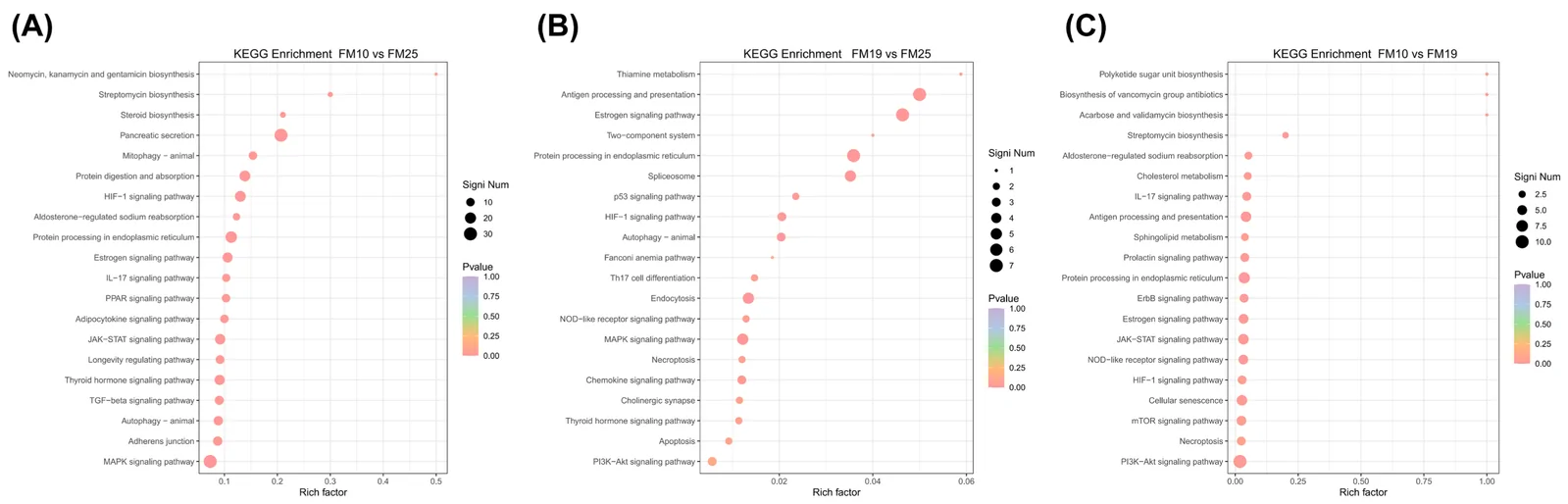
Kyoto Encyclopedia of Genes and Genomes pathway enrichment analysis of DEGs in the hepatic transcriptome of hybrid yellow catfish (*P. fulvidraco* ♀ × *P. vachelli* ♂) fed the FM10, FM19, and FM25 diets. (**A**–**C**) KEGG pathway enrichment of DEGs in the FM10 vs. FM25 (**A**), FM19 vs. FM25 (**B**), and FM10 vs. FM19 (**C**) comparisons. The x-axis represents the rich factor, calculated as the ratio of the number of DEGs assigned to a pathway to the total number of genes annotated to that pathway, whereas the y-axis shows the enriched KEGG pathways. Bubble size indicates the number of DEGs enriched in each pathway, and bubble color represents the enrichment *p*-value. A larger rich factor and a lower *p*-value indicate a higher degree of pathway enrichment.

Kyoto Encyclopedia of Genes and Genomes (KEGG) analysis revealed distinct functional patterns among the three comparisons. DEGs in FM10 vs. FM25 were mainly associated with stress responses (mitogen-activated protein kinase (MAPK) and hypoxia-inducible factor 1 (HIF-1) signaling, autophagy, and endoplasmic reticulum (ER) protein processing), endocrine regulation (thyroid hormone and JAK–STAT signaling), and metabolic homeostasis (peroxisome proliferator-activated receptor (PPAR) signaling and protein digestion and absorption). DEGs in FM19 vs. FM25 were primarily related to stress adaptation (HIF-1 and chemokine signaling, autophagy, and ER protein processing), cell survival and apoptosis (PI3K–Akt and p53 signaling and apoptosis), and hormonal regulation (thyroid hormone and estrogen signaling). In contrast, DEGs in FM10 vs. FM19 were predominantly associated with immune-inflammatory responses (NOD-like receptor, JAK–STAT, and interleukin-17 signaling), metabolic regulation (PI3K–Akt, mTOR, and HIF-1 signaling), hormonal regulation (estrogen signaling), and cellular homeostasis (cellular senescence and ER protein processing) (Figure 5A–C).

#### 3.6.3. Oxidative Stress and Immune Gene Expression

**Figure 6:**
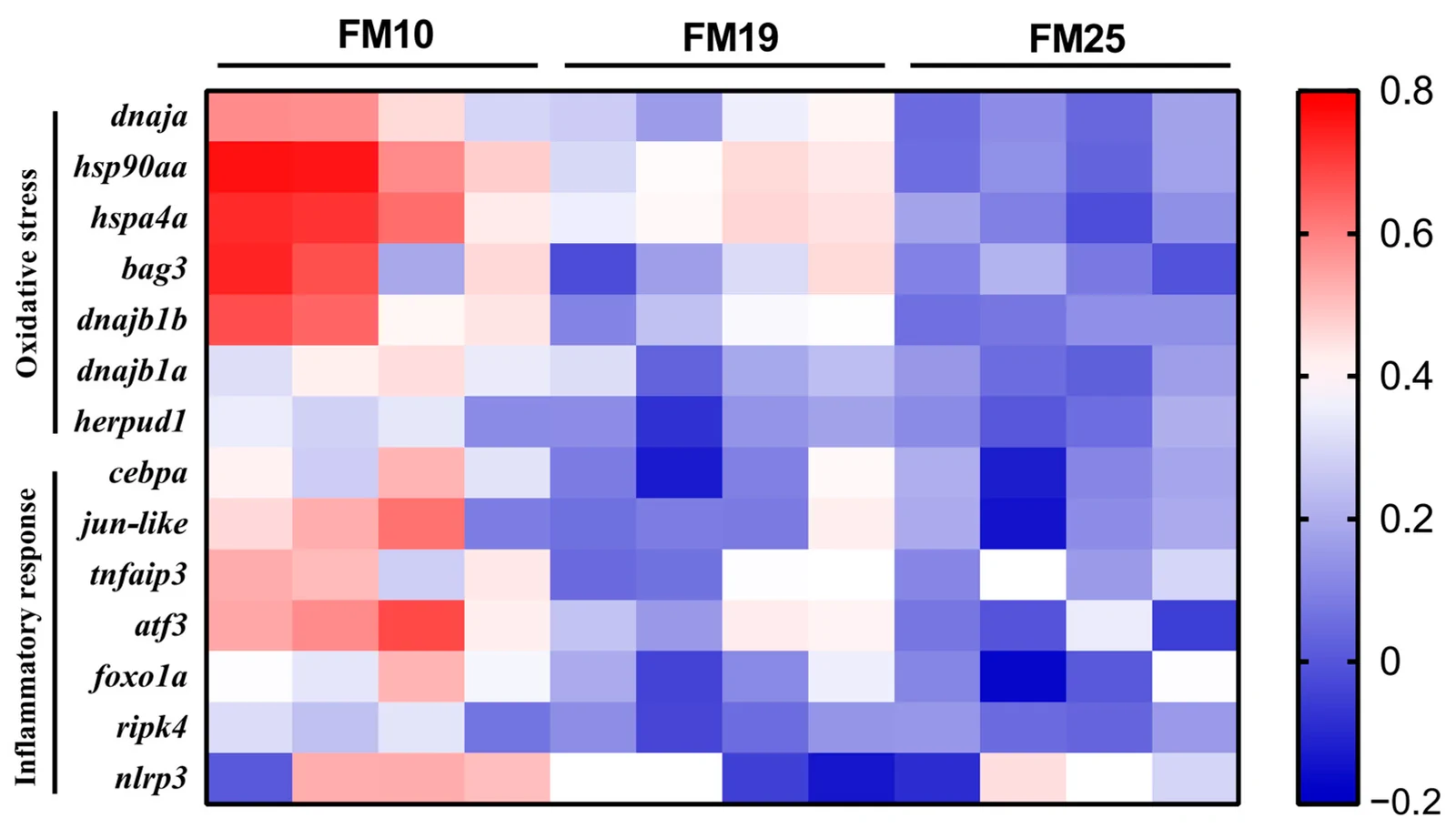
Expression of genes related to oxidative stress and inflammatory responses in the hepatic transcriptome of hybrid yellow catfish (*P. fulvidraco* ♀ × *P. vachelli* ♂) fed the FM10, FM19, and FM25 diets. The heatmap showed the relative expression levels of oxidative stress-related genes (*dnaja*, *hsp90aa*, *hspa4a*, *bag3*, *dnajb1b*, *dnajb1a*, and *herpud1*) and inflammatory response-related genes (*cebpa*, *jun-like*, *tnfaip3*, *atf3*, *foxo1a*, *ripk4*, and *nlrp3*) from the three dietary groups. Red and blue indicate relatively high and low expression levels, respectively.

In the liver of hybrid yellow catfish, the FM10 group generally exhibited higher expression levels of oxidative stress-related genes, including heat shock protein family genes (*hsp90aa*, *hspa4a*, *dnaja*, *dnajb1b*) and the co-chaperone-encoding gene *bag3*, as well as inflammation-related genes (*cebpa*, *jun-like*, *tnfaip3*, *atf3*, *foxo1a*, *ripk4*, *nlrp3*), than the FM19 and FM25 groups (Figure 6).

## 4. Discussion

In the present study, dietary replacement of FM with PBM exerted a level-dependent effect on the growth and feed utilization of hybrid yellow catfish. Growth performance initially increased and subsequently decreased with increasing FM replacement with PBM, whereas FCR showed the opposite pattern. These findings indicate that replacement of FM with PBM can improve feed utilization, whereas excessive replacement compromises growth. Comparable responses have been reported in cobia, rainbow trout (*Oncorhynchus mykiss*), and gibel carp (*Carassius auratus gibelio*) [13,23,24]. Neither HSI nor VSI differed among treatments, indicating that PBM inclusion did not significantly affect the relative liver or visceral mass. Although the CF differed among treatments, it showed no consistent relationship with either PBM replacement level or growth performance, indicating that CF was not a sensitive indicator of the growth response to dietary PBM under the present experimental conditions. Heterosis in fish is often associated with improved growth, feed utilization, physiological performance, and nutrient deposition relative to the parental strains [25,26]. Hybridization may also generate strain-specific differences in nutrient metabolism and dietary responses. Segmented linear regression based on WGR estimated an optimal FM replacement level of 27.4%, slightly higher than the 20.84% reported for juvenile yellow catfish [15]. These hybrid-specific physiological characteristics may partly account for the apparently greater tolerance of hybrid yellow catfish to PBM and the relatively high optimal FM replacement level observed in the present study. However, this interpretation requires validation through direct comparisons of hybrids and parental species under identical experimental conditions.

Serum biochemical indices provide important information on the nutritional status, hepatic function, and metabolic health of fish. In the present study, serum ALT and AST activities did not differ significantly among the dietary treatments, indicating that replacing FM with PBM in the tested range did not cause evident damage to hepatocyte integrity in hybrid yellow catfish. This result differs from the hepatic injury reported in loach fed high levels of PBM [18]. This discrepancy may be attributed to the maximum replacement level used in the present study, which may not have reached the threshold required to induce hepatocellular injury, as well as species-specific differences in the physiological adaptation to and utilization of PBM. In contrast, serum TG and TC concentrations differed significantly among the dietary treatments, suggesting that FM replacement with PBM affected lipid metabolism in a replacement-level-dependent manner. Changes in serum lipid concentrations may be related to differences in the fatty acid profiles. Compared with FM, PBM is generally characterized by lower levels of n-3 long-chain polyunsaturated fatty acids, and higher proportions of saturated and n-6 fatty acids. These differences in fatty acid profiles may modulate hepatic lipid metabolism and consequently alter TG and TC synthesis and secretion [27,28].

Digestive enzyme activities are commonly used as indicators of nutrient digestion and utilization in fish. In the present study, LPS and AMS activities were unaffected by dietary treatment, whereas intestinal trypsin activity declined significantly with increasing FM replacement with PBM, may reduce the capacity for protein digestion in hybrid yellow catfish. Similar reductions in digestive enzyme or protease activities have been reported in loach and juvenile yellow catfish fed diets containing high levels of PBM [15,18]. This response may be associated with differences between FM and PBM in amino acid composition, protein structure, and substrate availability, which may influence protease secretion or activity. Consistently, high soybean meal inclusion suppressed intestinal digestive enzyme activities in juvenile Asian red-tailed catfish (*Hemibagrus wyckioides*) [29], suggesting that excessive replacement of FM with alternative protein sources may compromise digestive function by altering dietary protein quality and digestibility.

Antioxidant capacity and disease resistance are key indicators of fish health and stress resilience. In the present study, hepatic and intestinal SOD activities generally decreased with increasing PBM inclusion, whereas hepatic CAT activity initially increased and then declined. This pattern suggests that moderate FM replacement may elicit a compensatory CAT response, while 60% replacement may overwhelm antioxidant regulation and impair the coordinated action of SOD and CAT [22,30]. Correspondingly, the significantly lower survival of the 60% replacement group at 96 h after *E. ictaluri* challenge indicated reduced disease resistance. Similar adverse effects of high FM replacement on antioxidant capacity have been reported in mandarin fish and Asian red-tailed catfish [19,29]. These effects may result from reduced dietary availability of FM-derived bioactive nutrients, such as taurine and nucleotides, together with impaired regulation of reactive oxygen species during the phagocyte respiratory burst, ultimately compromising pathogen clearance [14,31,32]. Collectively, excessive PBM replacement may weaken antioxidant defenses and disease resistance in hybrid yellow catfish.

Hepatic transcriptome analysis provided further insights into the response of hybrid yellow catfish to extensive replacement of FM with PBM. PCA clearly separated the FM10 group from the FM25 and FM19 groups, suggesting that replacing 60% of dietary FM with PBM substantially altered the hepatic transcriptional response. Furthermore, KEGG analysis showed that DEGs between the FM10 and FM25 groups were mainly enriched in the MAPK, JAK-STAT, and PPAR signaling pathways. The MAPK and JAK-STAT signaling pathways are closely involved in cellular stress and inflammatory responses. Enrichment of DEGs in these pathways therefore suggests that high FM replacement with PBM may alter hepatic stress- and inflammation-related signaling [33,34,35]. Consistent with this interpretation, transcriptomic studies in Hong Kong catfish (*Clarias fuscus*) have demonstrated extensive modulation of stress-, apoptosis-, antioxidant-, and immune-related pathways under thermal stress [36]. High dietary PBM inclusion has likewise been associated with oxidative stress, metabolic disturbances, and inflammation in white shrimp (*Litopenaeus vannamei*) [35]. PPAR signaling is a major regulator of fatty acid oxidation and triglyceride metabolism, and its differential regulation may partly explain the changes in serum lipid concentrations observed in the high-PBM group [37,38].

Heat shock proteins act as molecular chaperones that facilitate the refolding or removal of misfolded proteins and thereby maintain ER proteostasis [39]. The significant enrichment of the protein-processing pathway in the ER in the high-PBM group suggests disruption of hepatic protein-folding homeostasis and the induction of an ER stress response [40,41]. The concomitant upregulation of heat shock protein genes and the co-chaperone gene *bag3* may represent a compensatory response aimed at restoring proteostasis. However, the concurrent upregulation of the stress- and inflammation-related genes *cebpa*, *nlrp3*, and *atf3* suggests that this response was accompanied by inflammatory signaling. Similarly, dietary replacement of FM with a compound protein hydrolysate containing enzymatically hydrolyzed Sargassum altered ER stress-associated proteins and activated ER stress- and apoptosis-related pathways in white shrimp [42]. High dietary PBM inclusion has also been reported to disrupt immune-related gene expression, promote nuclear factor kappa B phosphorylation, and induce hepatopancreatic oxidative stress [35]. In Chinese perch (*Siniperca chuatsi*), extensive replacement of FM with a composite protein blend containing PBM similarly impaired hepatic metabolism and antioxidant status [43]. Collectively, these findings indicate that high-PBM replacement may reflect broad changes in hepatic metabolic and immune-related transcriptional networks, potentially through coordinated perturbation of ER proteostasis, inflammatory signaling, and apoptosis-related pathways.

Nevertheless, several limitations in this study should be acknowledged. The transcriptomic analysis was restricted to hepatic mRNA and was not supported by protein-level or functional validation; therefore, the identified pathways should be interpreted as correlative rather than causal. Intestinal transcriptomic analysis was not performed, although PBM replacement affected intestinal antioxidant and digestive enzyme activities. In addition, the intraperitoneal *E. ictaluri* challenge bypassed natural infection routes and may not fully reflect field conditions. Finally, the use of four biological replicates may have limited the detection of subtle transcriptional responses. Further studies using larger sample sizes, intestinal tissues, natural-exposure challenge models, and molecular or functional validation were warranted.

## 5. Conclusions

In the present study, moderate FM replacement with PBM (12–24% replacement) supported or improved growth performance and feed utilization, with the most favorable response observed in the FM19 diet, corresponding to 24% FM replacement. In contrast, high replacement levels of 48–60% were associated with reduced antioxidant and digestive enzyme activities, altered lipid-related responses, and lower disease resistance. Transcriptomic analysis further revealed that replacing 60% of dietary FM with PBM reshaped hepatic metabolic and immune networks by altering pathways related to ER stress, inflammation, and apoptosis. Based on broken-line regression analysis of weight gain rate, the optimal FM replacement level was estimated at 27.4%. This value should be interpreted as a growth-based estimate rather than a universal optimum for all physiological and health-related endpoints. Collectively, these findings provide physiological and molecular evidence supporting the development of low-FM diets for hybrid yellow catfish.

## Figures and Tables

**Table 1:** Formulation and proximate composition of experimental diets (% dry weight).

Items	Diet					
	FM25	FM22	FM19	FM16	FM13	FM10
Ingredients (%)						
Peruvian fish meal	25.00	22.00	19.00	16.00	13.00	10.00
Poultry by-product meal	15.00	18.24	21.51	24.73	27.97	31.26
Soybean meal	10.00	10.00	10.00	10.00	10.00	10.00
Corn gluten meal	8.00	8.00	8.00	8.00	8.00	8.00
Cottonseed protein	5.00	5.00	5.00	5.00	5.00	5.00
Wheat flour	25.00	25.00	25.00	25.00	25.00	25.00
Soybean oil	3.30	3.16	3.02	2.88	2.74	2.60
Fish oil	1.00	1.00	1.00	1.00	1.00	1.00
Soybean lecithin	1.50	1.50	1.50	1.50	1.50	1.50
Monocalcium phosphate	1.50	1.50	1.50	1.50	1.50	1.50
Microcrystalline cellulose	1.20	1.00	0.77	0.59	0.40	0.15
Choline chloride	0.50	0.50	0.50	0.50	0.50	0.50
Vitamin premix ^a^	1.50	1.50	1.50	1.50	1.50	1.50
Mineral premix ^b^	1.50	1.50	1.50	1.50	1.50	1.50
Lysine	0.00	0.07	0.13	0.19	0.25	0.31
Methionine	0.00	0.02	0.04	0.06	0.08	0.10
Threonine	0.00	0.02	0.03	0.05	0.06	0.08
Total	100.00	100.00	100.00	100.00	100.00	100.00
Proximate analysis (%)						
Crude protein	43.63	43.88	43.71	43.46	43.82	43.46
Crude lipid	10.76	10.23	10.64	10.72	10.45	10.92
Ash	11.14	11.39	11.21	11.06	11.26	11.45

^a^ Composition of vitamin premix (mg/kg diet): vitamin B_1_, 100; vitamin B_2_, 50; vitamin B_6_, 120; vitamin B_12_, 10; vitamin K_3_, 40; inositol, 1000; folic acid, 10; nicotinic acid, 200; retinyl acetate, 32; α-tocopherol, 240; ascorbic acid, 2000; riboflavin, 45; biotin, 3; cholecalciferol, 5; thiamin, 25. ^b^ Composition of mineral premix (mg/kg diet): MnSO_4_·H_2_O, 45; Na_2_SeO_3_ (1%), 20; MgSO_4_·7H_2_O, 1200; CuSO_4_·5H_2_O, 10; Ca(IO_3_)_2_ (1%), 60; CoCl_2_·6H_2_O (1%), 50; FeSO_4_·H_2_O, 80; ZnSO_4_·H_2_O, 5; Zeolite, 3485.

**Table 2:** Growth performance of hybrid yellow catfish (*P. fulvidraco* ♀ × *P. vachelli* ♂) fed diets with graded levels of FM replaced by PBM.

Items	FM25	FM22	FM19	FM16	FM13	FM10
SR (%)	100 ± 0 ^b^	100 ± 0 ^b^	100 ± 0 ^b^	100 ± 0 ^b^	99.75 ± 0.25 ^a^	100 ± 0 ^b^
IBW (g)	5.18 ± 0.01	5.17 ± 0.02	5.20 ± 0.007	5.18 ± 0.02	5.19 ± 0.01	5.18 ± 0.02
FBW (g)	33.68 ± 0.20 ^bc^	33.81 ± 0.32 ^bc^	34.96 ± 0.30 ^c^	34.36 ± 0.30 ^c^	32.62 ± 0.29 ^b^	30.52 ± 0.25 ^a^
WGR (%)	550.27 ± 3.27 ^b^	554.07 ± 7.45 ^b^	572.57 ± 5.89 ^c^	563.56 ± 5.20 ^c^	528.55 ± 4.89 ^b^	489.19 ± 6.96 ^a^
SGR (%/d)	3.34 ± 0.01 ^bc^	3.35 ± 0.02 ^bc^	3.40 ± 0.01 ^c^	3.38 ± 0.02 ^c^	3.28 ± 0.02 ^b^	3.17 ± 0.01 ^a^
CF (g/cm^3^)	1.77 ± 0.04 ^b^	1.75 ± 0.04 ^b^	1.70 ± 0.05 ^ab^	1.67 ± 0.10 ^a^	1.69 ± 0.03 ^ab^	1.67 ± 0.04 ^a^
HSI (%)	1.32 ± 0.08	1.41 ± 0.10	1.31 ± 0.08	1.43 ± 0.08	1.34 ± 0.09	1.39 ± 0.08
VSI (%)	7.87 ± 0.27	7.87 ± 0.37	7.81 ± 0.29	7.81 ± 0.21	7.83 ± 0.38	7.75 ± 0.22
FCR	1.19 ± 0.01 ^b^	1.22 ± 0.03 ^c^	1.13 ± 0.01 ^a^	1.14 ± 0.01 ^a^	1.22 ± 0.02 ^c^	1.18 ± 0.05 ^b^

Values in the same row with the same letter indicated no significant difference (*p* > 0.05; Tukey’s test). Abbreviations: SR, survival rate; IBW, initial body weight; FBW, final body weight; WGR, weight gain rate; SGR, specific growth rate; CF, condition factor; HSI, hepatosomatic index; VSI, viscerosomatic index; FCR, feed conversion ratio.

**Table 3:** Serum biochemical indices of hybrid yellow catfish (*P. fulvidraco* ♀ × *P. vachelli* ♂) fed diets with graded levels of FM replaced by PBM.

Items	FM25	FM22	FM19	FM16	FM13	FM10
ALT (U/L)	38.07 ± 1.67	36.94 ± 3.00	36.03 ± 3.06	42.96 ± 2.38	37.96 ± 2.55	40.14 ± 2.05
AST (U/L)	38.25 ± 1.67	38.74 ± 2.20	40.65 ± 2.14	40.15 ± 1.81	39.85 ± 1.98	36.22 ± 1.69
TG (mmol/L)	0.49 ± 0.04 ^b^	0.71 ± 0.04 ^a^	0.59 ± 0.05 ^ab^	0.72 ± 0.05 ^a^	0.54 ± 0.03 ^b^	0.72 ± 0.04 ^a^
TC (mmol/L)	48.10 ± 6.58 ^b^	51.52 ± 5.40 ^ab^	53.49 ± 7.24 ^ab^	56.98 ± 6.25 ^a^	47.26 ± 2.96 ^b^	47.58 ± 3.32 ^b^

Values in the same row with the same letter indicated no significant difference (*p* > 0.05; Tukey’s test). Abbreviations: ALT, alanine aminotransferase; and AST, aspartate aminotransferase; TG, triglycerides; TC, total cholesterol.

**Table 4:** Hepatic and intestinal antioxidant indices of hybrid yellow catfish (*P. fulvidraco* ♀ × *P. vachelli* ♂) fed diets with graded levels of FM replaced by PBM.

Tissue	Items	FM25	FM22	FM19	FM16	FM13	FM10
Liver	SOD (U/mg)	117.73 ± 4.12 ^a^	109.55 ± 5.03 ^ab^	111.87 ± 4.78 ^ab^	107.10 ± 3.60 ^ab^	107.98 ± 3.93 ^ab^	97.18 ± 3.92 ^b^
	CAT (U/mg)	39.71 ± 2.47 ^ab^	33.97 ± 0.91 ^b^	43.17 ± 1.93 ^a^	41.06 ± 2.76 ^ab^	38.17 ± 2.30 ^ab^	35.49 ± 2.65 ^ab^
	MDA (nmol/mg)	0.42 ± 0.02	0.45 ± 0.03	0.38 ± 0.04	0.37 ± 0.03	0.34 ± 0.03	0.35 ± 0.03
Intestine	SOD (U/mg)	129.70 ± 4.22 ^a^	124.80 ± 3.57 ^ab^	110.53 ± 3.16 ^bc^	120.17 ± 4.50 ^abc^	110.60 ± 3.50 ^bc^	109.62 ± 2.30 ^c^
	CAT (U/mg)	2.84 ± 0.62	1.94 ± 0.08	2.62 ± 0.39	1.91 ± 0.83	2.41 ± 0.78	1.75 ± 0.63
	MDA (nmol/mg)	0.42 ± 0.06	0.41 ± 0.05	0.39 ± 0.02	0.37 ± 0.02	0.40 ± 0.04	0.40 ± 0.07

Values in the same row with the same letter indicated no significant difference (*p* > 0.05; Tukey’s test). Abbreviations: SOD, superoxide dismutase; CAT, catalase; MDA, malondialdehyde.

**Table 5:** Activities of intestinal digestive enzymes in hybrid yellow catfish (*P. fulvidraco* ♀ × *P. vachelli* ♂) fed diets with graded levels of FM replaced by PBM.

Items	FM25	FM22	FM19	FM16	FM13	FM10
Trypsin (U/g)	3223.90 ± 453.00 ^a^	2990.70 ± 340.00 ^ab^	2251.35 ± 129.71 ^bc^	1953.50 ± 195.00 ^c^	1602.05 ± 345.00 ^c^	1759.22 ± 186.00 ^c^
LPS (U/g)	55.20 ± 16.67	44.86 ± 2.38	45.80 ± 4.56	38.73 ± 7.70	38.24 ± 15.30	35.40 ± 4.70
AMS (U/g)	3.16 ± 0.42	3.70 ± 0.40	2.49 ± 0.23	2.03 ± 0.36	3.37 ± 0.52	2.59 ± 0.45

Values in the same row with the same letter indicated no significant difference (*p* > 0.05; Tukey’s test). Abbreviations: LPS, lipase; AMS, α-amylase.

**Table 6:** Cumulative survival of hybrid yellow catfish (*P. fulvidraco* ♀ × *P. vachelli* ♂) challenged with *E. ictaluri* after feeding diets with graded levels of PBM replacing FM.

Group		FM25	FM22	FM19	FM16	FM13	FM10
**Time**	0 h	100.00 ± 0.00 ^a^	100.00 ± 0.00 ^a^	100.00 ± 0.00 ^a^	100.00 ± 0.00 ^a^	100.00 ± 0.00 ^a^	100.00 ± 0.00 ^a^
	6 h	100.00 ± 0.00 ^a^	100.00 ± 0.00 ^a^	98.33 ± 1.67 ^a^	98.33 ± 1.67 ^a^	98.33 ± 1.67 ^a^	95.00 ± 2.89 ^a^
	12 h	96.67 ± 3.33 ^a^	98.33 ± 1.67 ^a^	93.33 ± 3.33 ^a^	93.33 ± 3.33 ^a^	96.67 ± 3.33 ^a^	76.67 ± 1.67 ^b^
	24 h	90.00 ± 2.89 ^a^	90.00 ± 5.00 ^a^	86.67 ± 3.33 ^a^	80.00 ± 2.89 ^ab^	88.33 ± 6.67 ^a^	65.00 ± 2.89 ^b^
	48 h	83.33 ± 1.67 ^a^	78.33 ± 6.24 ^ab^	78.33 ± 3.33 ^ab^	70.00 ± 5.00 ^ab^	80.00 ± 4.41 ^ab^	56.67 ± 4.41 ^b^
	72 h	73.33 ± 4.41 ^a^	65.00 ± 7.26 ^ab^	66.67 ± 4.41 ^ab^	63.33 ± 4.41 ^ab^	63.33 ± 4.41 ^ab^	43.33 ± 6.24 ^b^
	96 h	63.33 ± 3.33 ^a^	56.67 ± 6.67 ^a^	60.00 ± 4.08 ^a^	56.67 ± 6.24 ^a^	51.67 ± 1.67 ^ab^	33.33 ± 3.33 ^b^

Values in the same row with the same letter indicated no significant difference (*p* > 0.05; Tukey’s test).

**Table 7:** Quality control of hepatic transcriptome data in hybrid yellow catfish (*P. fulvidraco* ♀ × *P. vachelli* ♂).

Sample	Clean Reads	Clean Bases	Q20 Bases Ratio (%)	Q30 Bases Ratio (%)	GC Content (%)
FM25_1	55,004,480	8,005,730,591	99.58%	98.35%	47.27%
FM25_2	46,895,482	6,837,754,197	99.29%	97.36%	46.89%
FM25_3	46,201,720	6,792,083,096	99.50%	98.02%	46.64%
FM25_4	55,511,288	8,142,794,278	99.57%	98.30%	47.00%
FM19_1	47,663,194	7,023,517,876	99.50%	98.03%	46.44%
FM19_2	37,100,572	5,454,077,724	99.54%	98.18%	47.07%
FM19_3	51,545,794	7,477,329,855	99.28%	97.40%	46.60%
FM19_4	57,495,384	8,464,969,169	99.46%	97.87%	46.57%
FM10_1	49,798,880	7,336,159,783	99.45%	97.84%	47.20%
FM10_2	48,563,722	7,144,610,018	99.54%	98.19%	47.23%
FM10_3	58,296,068	8,557,727,951	99.55%	98.23%	47.22%
FM10_4	45,766,874	6,742,798,634	99.50%	98.03%	46.90%

## Data Availability

The original contributions presented in this study were included in the article. Further inquiries can be directed to the corresponding author. The raw RNA-seq data generated in this study have been deposited in the NCBI Sequence Read Archive under accession number (SUB16452100).
